# The presence of adjacent others facilitates interpersonal neural synchronization in the left prefrontal cortex during a simple addition task

**DOI:** 10.1038/s41598-022-16936-3

**Published:** 2022-07-25

**Authors:** Naoki Miura, Satsuki Noguchi

**Affiliations:** Department of Information and Communication Engineering, Faculty of Engineering, Tohoku Institute of Technology, 35-1 Yagiyama Kasumicho, Taihaku-ku, Sendai, Miyagi 982-8577 Japan

**Keywords:** Cooperation, Social behaviour, Cognitive control

## Abstract

The hyperscanning technique, that is, simultaneous measurement of neural signals in more than one person, is a powerful research tool for understanding humans’ social interactions. In recent years, many studies have investigated interpersonal neural synchronization during various types of communication processes. However, there has been little focus on the impact of the presence of others without explicit social interaction, despite the mere presence of others having been suggested as influencing behavior. In this study, we clarify whether neural signals during a self-paced, repeated, addition task are synchronized when another individual is adjacent without direct interaction. Twenty pairs of participants were measured using a hyperscanning approach with near-infrared spectroscopy. The results show that interpersonal neural synchronization of the task-related signal in the left forehead region was enhanced under the condition of being adjacent to another participant. By contrast, a significant decrease in neural synchronization in the center of the forehead region, where increased neural synchronization is often reported in explicit communication, was observed. Thus, the results indicate that the adjacency of others modulates interpersonal neural synchronization in the task-related signal, and the effect on cognitive processing is different from that of explicit social interaction.

## Introduction

Humans usually interact with others in social life and these interactions can change the thoughts and behaviors of individuals. The effects can occur not only when there is explicit communication with others, but also when people are nearby, so that they are simply aware of each other. The latter effect is defined as social facilitation or inhibition, depending on the positivity or negativity of its effect^1,2^. Previous studies have reported that whether the presence of others facilitates or inhibits task execution depends on the characteristics of the task^3^: typically, the presence of others causes social facilitation in low-load tasks and social inhibition in high-load tasks. Therefore, cognitive processes for task execution and cognitive processes for awareness of others may interact with each other. In addition, if several people’s behaviors are socially facilitated or inhibited, common changes in cognitive processes may be occurring.

To elucidate changes in cognitive processing that occur during social interaction, it is important not only to analyze the behavioral and biological data of individuals but also to examine the interpersonal interactions among the individuals. Recently, hyperscanning techniques using near-infrared spectroscopy (NIRS) or electroencephalography have been widely used for examining the interpersonal synchronization of neural activity^4–6^. Previous studies have focused on explicit communicative group interaction such as cooperation^7–13^ and competition^14–16^ as the target of hyperscanning. In these studies, fluctuations in interpersonal neural synchronization are observed in a periodic band matching the task interval of repeated trials^8,11,12^. These findings indicate that social interactions with others influence the cognitive activities involved in specific task execution. However, neuronal synchronization of implicit social interactions has received less attention.

As well as explicit communication, the effects of implicit social interactions with others on neural activity can also be analyzed. A multi-person NIRS hyperscanning study reported that steady beat sounds induced synchronization of frontopolar activities and gait patterns across individuals during group walking^17^, that is, the neural signals of more than one person were synchronized even when cooperation was induced by external factors. Accordingly, if there is an implicit social interaction that arises from awareness of others, pronounced neural synchronization should be observed due to the fluctuations in brain activity caused by the execution of cognitive tasks. In addition, if the cognitive process of task execution is homogeneous after excluding the presence of others, the neural synchronization should be distinguished from the signal changes induced by the task. A functional magnetic resonance imaging study of social facilitation by the observer effect reported enhanced functional connectivity between cortical regions related to reward computation and behavioral motivation due to the presence of an observer^18^. Thus, if other people sharing a place were made aware of each other, their neural activity would be coordinated even if this is not directly related to their behavior.

In the present study, we used NIRS hyperscanning technique to investigate whether neuronal synchronization between two participants is enhanced when they perform a task adjacent to each other, even without explicit interaction. For this purpose, NIRS signals were measured simultaneously while both participants performed a simple addition task either adjacent to one another or individually. The brain regions whose NIRS signals were measured were the left lateral prefrontal, which is associated with execution of addition tasks^19,20^, and the frontopolar, where communication-related neural synchronization has been reported^8,11,13,17^. The effect of adjacent others on the interpersonal synchronization of neural signals was analyzed using the wavelet transform coherence (WTC) of the simultaneously measured NIRS signals.

## Results

### Behavioral data

Table 1 summarizes the mean (standard deviation) reaction time and correct response rate for the addition task for all participants in each of the adjacent and solitary conditions. Paired t-tests showed that there were no significant differences in task performance between the adjacent and solitary conditions in either reaction time (t(39) = − 0.3088, *p* = 0.7591, r = 0.0488) or correct response rate (t(39) = 0.4545, *p* = 0.6520, r = 0.0718).

**Table 1:** Mean (standard deviation [SD]) reaction time and correct response rate for the adjacent and solitary conditions.

Condition	Mean (SD) reaction time [s]	Mean (SD) correct response rate
Solitary	1.24 (0.32)	0.96 (0.04)
Adjacent	1.25 (0.35)	0.96 (0.04)

### Wavelet transform coherence analysis for neural synchronization

**Figure 1:**
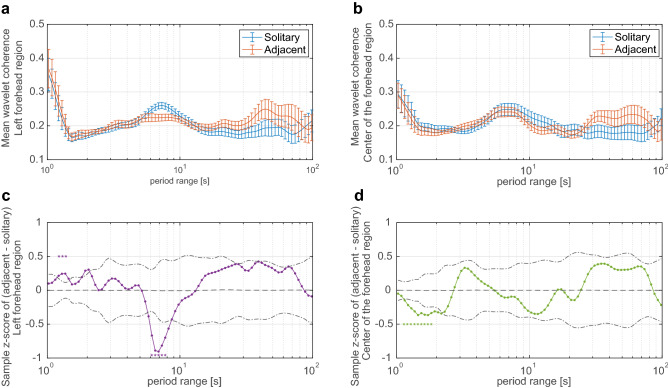
Comparison of interpersonal neural synchronization between adjacent and solitary conditions. Time-averaged wavelet transform coherence (WTC) of the neural signal under each condition for the (**a**) left and (**b**) center of the forehead. Each error bar indicates the standard error of the mean WTC for each pair. Sample z-score of between-condition difference (adjacent–solitary) for each period range for the (**c**) left and (**d**) center of the forehead. The asterisks indicate period ranges that showed statistically significant differences, and the dashed and long dashed dotted lines indicate the mean and ± 2 SD ranges of sample z-scores for the 10,000-permutation data.

To investigate the reciprocal effects of the presence of neighboring individuals on brain activity, the degree of interpersonal neural synchronization was examined by WTC analysis. Figure 1 shows a comparison of average WTC values for each of the 80 period ranges within the overall range of interest (i.e., 1–100 s) under the adjacent and solitary conditions. Table 2 summarizes the average WTC values and their statistics from permutation test by channels and period ranges. Significant increase in the average WTC values was observed under the adjacent condition compared to the solitary condition in three period ranges of 1.22 to 1.37 s on the left side of forehead channel. By contrast, significant decrease in the average WTC values was observed under the adjacent condition compared to the solitary condition in nine period ranges of 1.15 to 1.83 s on the center of forehead channel, and five period ranges of 6.14 to 7.74 s on the left side of forehead channel.

**Table 2:** Results of permutation test on the average WTC values for each measurement channel and condition.

Period range [s]	Left forehead			Center of the forehead		
	z-score	adjusted q	r	z-score	adjusted q	r
[1.03–1.09]*	–	n.s	–	–	n.s	–
1.15	0.1580	n.s	0.0250	− 0.1485	0.0420	0.0417
1.22	0.2151	0.0420	0.0167	− 0.2181	< 0.0000	0.0501
1.29	0.2446	0.0018	0.1419	− 0.2661	< 0.0000	0.2003
1.37	0.2465	0.0018	0.2170	− 0.3112	< 0.0000	0.2337
1.45	0.1718	n.s	0.1419	− 0.3662	< 0.0000	0.3423
1.53	0.0875	n.s	0.0334	− 0.3368	0.0048	0.3256
1.63	0.0961	n.s	0.1169	− 0.3607	0.0271	0.3256
1.72	0.1277	n.s	0.1587	− 0.3666	0.0271	0.2838
1.83	0.1952	n.s	0.2087	− 0.3356	0.0480	0.1920
[1.94–5.80]*	–	n.s	–		n.s	–
6.14	− 0.6679	< 0.0000	0.5677	− 0.0850	n.s	0.1503
6.51	− 0.8902	< 0.0000	0.6929	− 0.1070	n.s	0.1503
6.90	− 0.9047	< 0.0000	0.7012	− 0.1054	n.s	0.1419
7.31	− 0.8166	0.0072	0.6261	− 0.0929	n.s	0.0751
7.74	− 0.7000	0.0420	0.5927	− 0.0969	n.s	0.0501
[8.20–90.31]*	–	n.s	–	–	n.s	–

Adjusted q indicates statistical significance corrected for multiple comparisons with false-discovery rate; and, r indicates an effect size calculated using Wilcoxon signed-rank test. *: Period ranges [1.03–1.09], [1.94–5.80], and [8.20–90.31] includes 2, 20, and 44 period ranges of different duration, respectively.

### Condition-specific changes in neural signals

In addition to analysis of neuronal synchronization, we analyzed condition-specific neural signal changes and their relationship to task performance. Analysis of variance for task-related changes in the neural signal showed no significant main effect or interaction between the conditions, channels, and sex (main effect of condition: F(1,38) = 0.3371, *p* = 0.5649, generalized *η*^2^ = 0.0010; main effect of channel: F(1,38) = 0.0514, *p* = 0.8219, generalized *η*^2^ = 0.0006; main effect of sex: F(1,38) = 0.0144, *p* = 0.9052, generalized *η*^2^ = 0.0001; interaction between conditions and channels: F(1,38) = 1.5308, *p* = 0.2236, generalized *η*^2^ = 0.0051; interaction between sex and conditions: F(1,38) = 0.3371, *p* = 0.3832, generalized *η*^2^ = 0.0023; interaction between sex and channels: F(1,38) = 0.6352, *p* = 0.4304, generalized *η*^2^ = 0.0072; interaction between three factors: F(1,38) = 0.0390, *p* = 0.8444, generalized *η*^2^ = 0.0001).

**Figure 2:**
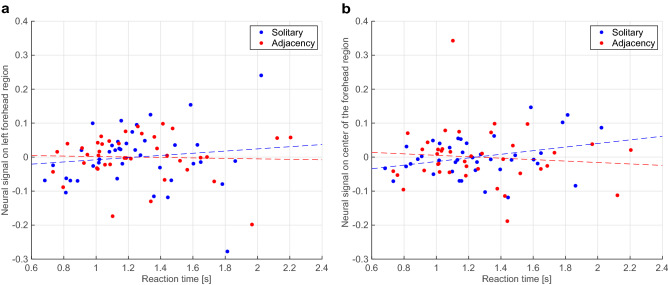
Relationship between reaction time and task-related change in neural signal under each condition for the (**a**) left and (**b**) center of the forehead. Note that the effect of participants’ sex was excluded from the change in each neural signal. The dashed line indicates the regression line.

Figure 2 shows a scatter plot of the reaction time and task-related change in neural signal under each of the adjacent and solitary conditions. The correlation coefficients for each combination are summarized in Table 3; no significant correlation was found between the reaction time for each adjacent and solitary task and the NIRS signal change observed for that task in each channel.

**Table 3:** Results of correlation analysis between reaction time and task-related signal changes for each measurement channel and condition.

Measurement channel	Condition	Correlation coefficient	*p*
Left forehead	Solitary	0.1183	0.4669
	Adjacent	− 0.0367	0.8222
Center of the forehead	Solitary	0.2980	0.0618
	Adjacent	− 0.0907	0.5778

*p* indicates the probability that the correlation coefficient is not significantly different from 0 (two-sided test). Because four correlation coefficients were obtained, the significance level corrected for a multiple comparison was set at *p* < 0.0125.

## Discussion

We investigated whether interpersonal neural synchronization of prefrontal activity occurs when performing a simple addition task adjacent to others in the absence of explicit interaction. The average WTC values of the left forehead region on specific period ranges were significantly increased under the adjacent condition compared to the solitary condition. On the other hand, significant decreases in the average WTC values between the adjacent and solitary conditions were observed at center of the forehead on similar period ranges; similarly, decreases in the average WTC values with longer period ranges were observed in the left forehead region. In addition, there were no differences in task-related activity between conditions or relationships with behavioral data.

This increase in interpersonal neural synchronization in the left frontal region suggests that the cortical activities associated with task execution of two adjacent individuals is modulated by each other’s mere presence. The period range with a significant increase in synchronization was 1.22–1.37 s, which is roughly consistent with the sum of the average reaction time for the adjacent condition and inter-trial interval. Previous studies have reported increased interpersonal neural synchronization over period ranges corresponding to trial duration^7,8,12^, and the left prefrontal region has been found to play a key role in numerical calculations^19,20^. Thus, the results indicate a synchronization of cognitive activities between a pair of participants involved in performing the addition task. Furthermore, the experimental task did not ask for explicit communication between the pair of participants under the adjacent condition, indeed, they were asked not to communicate during measurements, therefore participants were seated next to each other but were working on the task independently. Thus, we can interpret that awareness of adjacent individuals, even in the absence of explicit communication, triggers the interpersonal neural synchronization. These findings extend previously reported findings of interpersonal neural synchronization induced by explicit communicative interactions^7–16^ and external interactive triggers, such as rhythmic sounds^17^.

Neural synchronization by adjacency with others may modulate changes in regional signal fluctuations independent of the mental workload for task execution. Although there was an increase in interpersonal neural synchronization in the left forehead region, no correlation was observed between task-related signal enhancement and reaction time under adjacent and solitary conditions. In addition, ANOVA for task-related NIRS signals showed no significant difference between conditions. The behavioral data also indicated that participants had similar reaction times, suggesting that there were no significant differences between the conditions in terms of mental workload. Because the experimental task consisted of self-paced repetition of simple addition, reaction time may have reflected the subjective difficulty performing mental arithmetic. Task difficulty of mental arithmetic is related to NIRS signals in the left prefrontal cortex^21^. Taken together, the present findings suggest that the presence of others itself did not affect the magnitude of task-specific signal changes that reflect the mental workload for the addition task. By contrast, neural activity in this region seems to fluctuate depending on the cognitive state in which the task is addressed. It has been suggested that activity in the left prefrontal cortex reflects changes in behavioral tactics to respond to additional rules as well as task difficulty^22^. The period ranges of increased WTC values were short, considering the hemodynamic response measured by NIRS signal ^23^. Thus, the source of this variability is presumably the superimposed time-series signal induced by engaging in repeated task trials rather than the brain activity induced by a single trial. Thus, variability reflects the cognitive readiness to engage in the task, i.e., awareness of others. Based on these findings, we conclude that the presence of an adjacent individual produced a change in the cognitive state of task engagement, which affected NIRS signal independent of the mental workload. Furthermore, significant decrease of interpersonal neural synchronization was observed in the periodic ranges around 6 s. This peripheral period range is known to be related to respiration^24^ and Mayer wave^25^, and a similar, though not statistically significant, suppression was observed in the center of the forehead channel. Thus, it is possible to consider that some fluctuation in physiological state caused by the presence of other may have influenced the results.

In contrast to the neural signal in the left forehead region, significant decreases in interpersonal neural synchronization in similar period ranges were observed in the center of the forehead, or between the task-related NIRS signal and behavioral data. The frontal pole region is involved in social interaction^26^. A significant increase in NIRS signal has also been reported during face-to-face conversations^27^. Several hyperscanning studies have reported that social interaction increases interpersonal neural synchronization in the frontopolar region^8,13,17^. This discrepancy between regions is presumably due to differences in interactions with neighboring individuals in the experimental task. This experiment did not require explicit cooperation between pairs in performing the experimental task because each participant performed the simple addition task individually. In addition, the participants were instructed not to communicate during the measurement. Thus, the difference between adjacent and solitary conditions was the presence of adjacent others and not the presence of communicative behavior between participants. Based on these facts, we suggest that the lack of explicit interaction with adjacent others was the cause of decreased interpersonal neural synchronization in the frontopolar region. One possible interpretation is that there may have been an implied competitive relationship between adjacent others. Direct comparison of explicit cooperative and competitive tasks reported increased neural entrainment in cooperative tasks^7,14^. Although the lack of differences in task performance between conditions would not allow directly addressing this issue, it would suggest that there may have been some fluctuations in internal social cognition.

A limitation of the present study is that the effect of an adjacent participant on cortical activity could not be segregated from the behavioral data as to whether it was due to social facilitation or inhibition. Previous meta-analytic studies have reported facilitatory or inhibitory effects on reaction time and correct response rates depending on task difficulty, e.g., the presence of others often had a positive effect for both reaction time and task accuracy on simple tasks^3^. However, there were no significant differences between the adjacent and solitary conditions in either reaction time or correct response rate. This might be because the addition task was too simple to cause a behavioral difference. The average correct response rate was 0.96 under both conditions, suggesting that the difference between conditions was not revealed because of a ceiling effect. Similarly, in terms of reaction time, we can speculate that individual differences such as fluctuations caused by fatigue or habituation were greater than the social facilitating and inhibitory effects because the task involved rapidly repeated trials. Thus, we cannot distinguish whether the interpersonal neural synchronization observed in the left forehead region was the result of social facilitation or inhibition. However, we can assert that this synchronization was caused by adjacency with others.

The present findings had certain limitations related to participants' characteristics and relationships. Some hyperscanning studies involving explicit cooperation tasks have suggested that sex combinations of participant pairs may affect interpersonal neural synchronization^8,11^; these studies have reported a tendency for male–male pairs to have a higher degree of synchrony than other pairs. Because many of the participant pairs in the study were male–male, the interpersonal neural synchronization might be weaker if only other pair combinations are examined. In addition, social relationships between pairs may also be an important factor. A previous psychological study has reported that social status affects performance on the joint action task^28^, and neural synchronization during cooperative tasks was enhanced in pairs of lovers^12^. Because it is difficult to evaluate a wide range of social relationships, the present study only recruited university student pairs who were acquaintances, and thus did not include pairs who had never met before, pairs with closer relationships (e.g., lovers and family members), and pairs with different social statuses. In addition, subjective preference for conditions that reflect personality differences might also influence neural synchronization, although there were no differences in task performance. Therefore, future studies should take these relationships into account to enhance our understanding of group dynamics.

In conclusion, interpersonal neural synchronization in the left prefrontal region during the addition task was facilitated while that in the frontopolar region was inhibited by the presence of an adjacent individual without explicit communication between individuals. The occurrence of this synchronization was not directly related to the magnitude of task-related activity, and the signal components in specific period ranges were modulated during task execution. Thus, awareness of adjacent others affects task-related NIRS signal fluctuation despite the absence of explicit communication.

## Materials and methods

### Participants

A total of 40 healthy university students (34 males and 6 females; mean age = 21.2, standard deviation = 1.1, range = 20–25 years) participated in this experiment. All participants had normal or corrected-to-normal vision. Each experimental group consisted of one pair of participants. When recruiting, one participant was asked to bring one other participant. This is because social relations between the pairs were restricted to acquaintances within the university to control for such relations. Hence, the participant pairs were friends, or members of the same laboratory or hobby club, and all pairs’ members had met each other before. The distribution of sex combinations consisted of 16, 2, and 2 male–male, male–female, and female–female pairs, respectively. All participants provided written informed consent in response to an experimental protocol approved by the Research Ethics Committee of Tohoku Institute of Technology, Japan, and the experiments were performed in compliance with the Declaration of Helsinki.

### Experimental task

**Figure 3:**
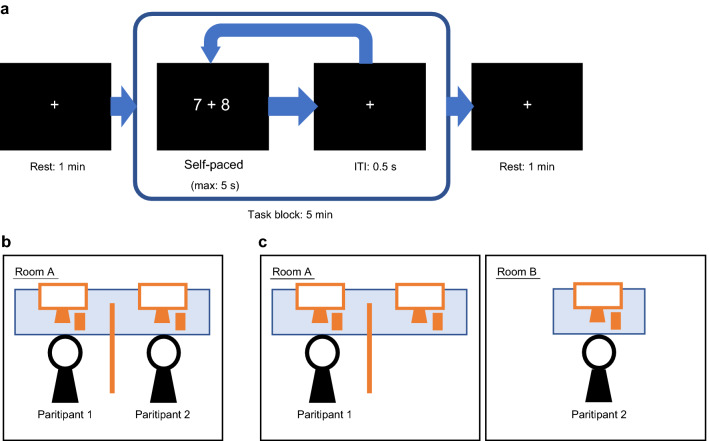
Overview of experimental design. (**a**) Timeline of each experimental run. The trials proceeded at each participant’s own pace, with a maximum duration for each of 5 s; the inter-trial interval was set at 0.5 s, and the duration of the trial block was 5 min. (**b**) Experimental environment of the adjacent condition. Each pair of participants performed the experimental task next to each other in the same room. (**c**) Experimental environment of the solitary condition. One of the participants was moved to another room, and both performed the experimental task simultaneously.

Figure 3a provides an overview of the experimental task. The experimental task was programmed using PsychoPy software^29^ and consisted of one-digit addition. To analyze temporal fluctuations of the time-series NIRS signal while performing the task, the experimental task was set in a block design, with short intervals to keep the participants busy during the task. In each trial, two single-digit numbers were displayed in the center of a screen with a plus sign between them. Participants were instructed to calculate the displayed formula and respond with the single-digit result using the numeric keypad as quickly as possible. Although it is necessary to exclude carryover to the tenth place, the task used in the present study was a simple cognitive task that only requires repetition of the addition result. They were also instructed to avoid speech and large body movements during measurements. The maximum duration of each trial was 5 s and the inter-trial interval was 0.5 s, however, each trial was terminated by the participant’s response, so participants performed the task at their own pace. The trials were grouped into a single task block, with resting periods before and after it. During a resting period, a fixation cross was displayed in the center of the screen, and participants were instructed to gaze at it. The duration of the task block was 5 min and the duration of the resting period was 1 min, thus the measurement time of one experimental run was 7 min.

Two conditions were prepared to examine the influence of adjacent partners on task execution. Under the adjacent condition, one pair of participants were seated next to each other in the same room and performed the task using an LCD monitor and numeric keypad in front of each of them (Fig. 3b). A semitransparent panel was placed between the participants. Thus, the subjects could not see each other’s monitors but could hear key presses during the task. Under the solitary condition, one member of the participant pair was moved to another room, and they both performed the task on the devices in their respective rooms (Fig. 3c). Under both conditions, the tasks for both partners were adjusted to begin simultaneously. To eliminate the influence of observer effects, the experimenter hid behind a partition placed behind the participants during measurements. The order of the adjacent and solitary conditions was randomly determined for each participant pair to control for order effects. Instruction and short-term practice (20 s of a practice task then 5 s of resting) were performed before the experiment began in the same environment as the adjacent condition.

### NIRS measurements

Signals in the region of the forehead were recorded using a wearable, two-channel, continuous-wave, NIRS device (HOT-2000; NeU Corporation, Tokyo, Japan). One measurement channel each was placed in the center of the forehead and left temple in the region of the forehead. When attaching the device to the participant, the measurement channel in the center of the forehead overlapped the Fpz site in the ten percent electrode system^30^, and the device position was adjusted slightly to avoid pain for the participant. In each measurement channel, shallow (source–detector distance = 1 cm) and deep (source–detector distance = 3 cm) signals indicating estimated concentration changes in total hemoglobin were measured at a sampling frequency of 10 Hz.

### NIRS data preprocessing

Data preprocessing was performed following Nozawa et al.^13^. All preprocessing and statistical analyses were performed using MATLAB (MathWorks, Natick, MA, USA) and R software^31^. First, the data series for each run was extracted from the time-series of shallow and deep signal data, including the task and the resting periods before and after it. Thus, each time series data contained 420 s of data. Missing data caused by unstable Bluetooth communication were interpolated linearly from the data before and after them. Second, drift components in the data series were removed using a third-order polynomial function. Third, motion artifacts were corrected using wavelet-based motion artifact reduction implemented in HomER3 software^32^ with the threshold parameter for excluding outliers set at 5.0. These procedures were performed separately for shallow and deep signals. Finally, the neural signal was obtained by excluding the effect of skin blood flow signal from the deep signal. We used eigenvector-based skin blood flow attenuation algorithms^33^. Eigenvectors were computed from the four measured signals (shallow and deep signals from the left and center of areas of forehead) in the first resting period (60 s). Then, the filter was constructed from the first two components based on the findings of Keshmiri et al.^34^. The time series of neural signal for each channel was then obtained by applying the filter to the deep signal.

### Wavelet transform coherence analysis for interpersonal neural synchronization

To examine the influence of neighboring individuals on their neural activity during task execution, we analyzed interpersonal neural synchronization between each pair of participants who performed the adjacent and solitary tasks together. We calculated the magnitude-squared WTC value of the preprocessed time series data of the neural signal using the wavelet toolbox for MATLAB^7^. In other words, WTC values were calculated between time series of neural signals at the same channel position for each pair member. Then, WTC values were calculated for each of the channel position (left and center of forehead) and each of the adjacent and solitary conditions. Thus, four WTC value matrices (rows indicate period ranges and columns indicate time points) were obtained from each pair. Because we presumed that the brain regions producing differences in blood flow between the two channels were themselves different, we did not consider signal synchronization across measurement channels; only the difference between two measurement channels was used for multiple comparisons.

To evaluate the difference in neural synchronization between the conditions, we performed nonparametric permutation tests of the time-averaged WTC values. Because we focused on neural synchronization induced by short-term task-specific signal fluctuation as well as across longer-term task-block effect, we applied a testing procedure for fine timescale bins^13^. The range of periods chosen was 1–100 s (0.01–1 Hz) to cover the entire frequency range of previous NIRS hyperscanning studies, and the time-averaged WTC values from 80 period ranges were selected for the analysis. The time-averaged WTC value for each period range during task period was calculated for each of the adjacent and solitary conditions and two measurement channels, excluding data in the cone of influence. The difference in time-averaged WTC value of adjacent minus solitary conditions was calculated for each pair of participants for each period range, and the sample z-score for the entire pair was calculated for each period range and measurement channel.

To test the difference between conditions, we created 10,000 permutation data points. These permutated data preserved pair-to-pair dependencies, i.e., data points were created by randomly swapping between conditions for each pair. The null distribution of the test statistic was obtained from the permutated data, because if the presence of neighbors does not affect neural synchronization in the adjacent condition, the difference in time-averaged WTC values between the adjacent and solitary conditions should be zero. The significance of the original difference in the time-averaged WTC value between adjacent minus solitary conditions was tested against that distribution for each period with a two-sided test. False-discovery rate (FDR) adjustment^35^ was used for multiple comparisons of period ranges and measurement channels. Because there were 80 period ranges of interest and 2 measurement channels, multiple comparisons were made with a total of 160 permutation tests. The significance level was set at q < 0.05.

### Analysis for task-related changes on time-series NIRS signals

To identify the relationship between the influence of cortical blood flow changes from adjacent others and neuronal synchronization, we also analyzed the task-related changes in neural signals. The average task-related change in neural signal relative to the resting period just before the task block for each condition was computed for each channel from the preprocessed neural signals. We performed repeated measures analysis of variance with conditions (adjacent or solitary) and measurement channels (center or left side of forehead) as within-subject factors, and participants’ sex as a between-subject factor.

To further clarify the relationship between task performance and task-related changes in neural signal for each condition, we performed correlation analysis between the task-related change in neural signal per channel and reaction time for each condition. To exclude sex differences in NIRS signal changes^36,37^, a correlation analysis was performed between the residuals obtained from multiple regression analysis of NIRS data with sex, encoded by one-hot encoding, as explanatory variables, and the reaction time. Because there were four combinations of measurement channels and conditions, the significance level of the test for non-correlation was set at *p* < 0.0125, corrected for multiple comparison by the Bonferroni method.

## Data Availability

The datasets generated during and/or analyzed during the current study are available from the corresponding author on reasonable request.
